# Determination of chromium in biological materials by radiochemical neutron activation analysis (RNAA) using manganese dioxide

**DOI:** 10.1007/s10967-016-4896-0

**Published:** 2016-06-13

**Authors:** Iga Kużelewska, Halina Polkowska-Motrenko, Bożena Danko

**Affiliations:** Laboratory of Nuclear Analytical Methods, Institute of Nuclear Chemistry and Technology, Dorodna 16, 03-195 Warsaw, Poland

**Keywords:** Chromium, Radiochemical neutron activation analysis, Radiochemical separation, Manganese dioxide, Inorganic resin

## Abstract

A procedure based on radiochemical neutron activation analysis was developed for the determination of chromium in biological materials. The procedure consists of irradiation of reference and test samples in a nuclear reactor, microwave sample digestion, selective and quantitative radiochemical separations of chromium and gamma-ray spectrometric measurement of ^51^Cr. Separation of chromium from the accompanying elements was done on the column packed with inorganic resin MnO_2_ Resin. Distribution coefficients of Cr, Zn, Co, Cs and Sc were determined in the system: MnO_2_ Resin—HCl, HNO_3_ and H_2_SO_4_. Accuracy of the procedure was checked by analysis of certified reference materials.

## Introduction

Chromium is an element belonging to the group of potentially toxic metals. It is a pollutant, mainly derived from human activities such as leather tanning, electroplating, wood preserving and the metallurgy industry [1–3]. Toxicity of chromium compounds strongly depends on its oxidation state [4]. Chromium can exist in several oxidation states; the most important are Cr(III) and Cr(VI). Trivalent chromium is an essential micronutrient. It is a part of the enzymes affecting the metabolism of glucose, the so-called glucose tolerance factor (GTF). Chromium deficiency can cause diabetes and cardiovascular diseases [5, 6]. Hexavalent chromium has strong oxidizing properties. It can penetrate biological membranes and has a toxic effect on humans, animals and plants. Excessive exposure to Cr(VI) can cause skin damage, respiratory problems, cancer of the kidneys or liver [7, 8]. Therefore, the content of chromium in environmental and food materials should be constantly monitored.

Accurate determination of traces of Cr in biological materials can be a serious problem and challenge. Different methods are applied, including atomic absorption spectrometry (AAS), inductively coupled plasma optical emission spectrometry (ICP-OES), inductively coupled plasma mass spectrometry (ICP-MS), spectrophotometry, chemiluminescence and neutron activation analysis (NAA). Most of the analytical techniques suffer from a number of different factors affecting the accuracy of chromium determination, such as contamination, volatility losses, incomplete dissolution, multiple oxidation states/chemical species, absorption/adsorption, interferences, incomplete separations or matrix effects [9–12]. The results of interlaboratory comparisons (ILC) frequently show the large discrepancies in chromium concentrations. This is also evidenced by the results of ILCs organized recently by the Institute of Nuclear Chemistry and Technology (INCT). The spread of results obtained for Cr (0.071–19.517 mg kg^−1^) supplied by the ILC participants in the case of material of plant origin, INCT-OBTL-5 made it impossible to assign a certified value [13]. Among the above-mentioned techniques applied for the determination of chromium, NAA has the highest metrological quality [14] and plays an important role in the certification of reference materials [15, 16]. NAA can be in two modes: instrumental neutron activation analysis (INAA) and radiochemical neutron activation analysis (RNAA).

RNAA is a combination of neutron activation with a post-irradiation separation of the determined element and gamma-ray spectrometry measurement [12, 13, 17, 18]. This method was used for the development of the ratio primary reference measurement procedures for the determination of cadmium, cobalt, nickel, molybdenum, uranium, iron, arsenic and selenium in biological materials [17–21]. The aim of this study was to develop a RNAA procedure for the determination of total Cr at trace levels in biological samples.

## Experimental

### Reagents, radioactive tracers

MnO_2_ Resin 100–200 mesh (Eichrom Technologies LLC) was used. A standard solution of Cr(VI) (10 mg g^−1^) was prepared from analytical grade K_2_Cr_2_O_7_ by dissolution in water. Chromium standards for irradiation were prepared by weighing aliquots of the standard solution in PE capsules (Type “V”, Vrije Universiteit, Biologisch Laboratorium, The Netherlands) and evaporating them to dryness before encapsulation. The following radioactive tracers were used: ^134^Cs (T_1/2_ = 2.06 years), ^60^Co (T_1/2_ = 5.27 years), ^51^Cr (T_1/2_ = 27.7 days), ^46^Sc (T_1/2_ = 83.8 days), ^65^Zn (T_1/2_ = 244 days). All tracers were prepared by neutron irradiation of spectrally pure oxides or salts (mostly nitrates) in a Polish nuclear reactor MARIA (neutron flux ~ 10^14^ cm^−2^ s^−1^). All reagents were of analytical grade. High purity water18 MΩ cm from Mili Q RG Ultra Pure Water System, Millipore Co. was used for the preparation of all solutions.

## Apparatus

Micro-analytical and analytical balances, Sartorius MC5 and Sartorius BP221S, were used to prepare standards and tracer for irradiation.

A high-pressure system Anton Paar 3000 was applied to digest samples.

Gamma-ray spectroscopic measurements were performed with the aid of the 180 cm^3^ HPGe well-type (Canberra) with associated electronics (Ortec) (resolution 2.09 keV for 1332 keV ^60^Co line, efficiency approximately 35 %), coupled to the multichannel analyzer TUKAN (NCNR, Świerk, Poland).

Glass columns of I.D. 0.50 cm were used in column experiments. The resin bed of 10 cm length was rested on a glass wool plug.

## Separation scheme and RNAA procedure

Biological samples (100–150 mg) and chromium standards (10 µg) in PE capsules were irradiated in nuclear reactor MARIA at thermal neutron flux of 10^14^ cm^−2^ s^−1^ for 50 min. After approximately 1 week of cooling, the samples were quantitatively transferred into Teflon vessels, and nonactive Cr carrier (50 µg) was added. All PE capsules were additionally washed with acid and washings were then added to the sample before mineralization process. Then the mineralization process was carried out in high pressure microwave system under controlled conditions, using a mixture of 6 mL of concentrated HNO_3_ and 2 mL of 30 % H_2_O_2_ and 1 mL of HF. The resulting solution was transferred into a Teflon vessel and evaporated to wet salts. The residue was dissolved in 2 mL of 0.01 M H_2_SO_4_. Finally, the obtained solution was introduced onto the top of the column filled with MnO_2_ Resin, 100–200 mesh (h = 10 cm, r = 0.25 cm). The column was washed with 15 mL 0.1 M HNO_3_ (elution of impurities, i.e. antimony, rubidium, zinc and cobalt). Next, the column was washed with 4 M HCl (elution of cesium and the rest of rubidium). The retained Cr(VI) was quantitatively eluted with 8 M H_2_SO_4_. The flow rate was maintained at 1.2–1.5 cm min^−1^. The chromium content was determined by gamma-ray spectrometry via the 320 keV line. The measurement time varied between 2000 and 20,000 s.

## Results and discussion

NAA is one of the best analytical techniques available for determination of trace level of chromium in a variety of materials. The half-life of the radionuclide ^51^Cr (T = 27.7 days) gives the opportunity to apply both versions of NAA: instrumental (nondestructive) and radiochemical. However, Cr is considered to be a difficult element for instrumental neutron activation analysis due to influence of a high radioactivity of the biological matrix caused mainly by bremsstrahlung of ^32^P and Compton continuum from high activities of radionuclides emitting high energy gamma-rays [12]. Those effects can be eliminated by the separation of chromium from others elements. Furthermore, polyethylene capsules, commonly used as canning material, generate a signal of ^51^Cr (320 keV) which interferes with the signal generated by the investigated sample. PE capsules used in this work contained 0.66–2.39 μg Cr. Therefore, the Szilard–Chalmers effect was examined and the effect of nuclear recoil was found negligible. Also, the irradiated samples were removed from the capsules before processing. Another problems can be caused by possible spectral and nuclear interferences. Therefore possible influence of interfering nuclear reactions were considered. It was found that only reaction ^54^Fe(n, α) ^51^Cr can potentially have a significant impact on the result of chromium determination. Under applied irradiation conditions, 20.7 μg of Cr is formed from 1 g of Fe. Taking into account the content of iron in most biological materials, the influence of the above-mentioned reaction can be disregarded. In the case of ultratrace levels, it is most often necessary to perform radiochemical separations to isolate chromium from other elements that can interfere directly via gamma-ray overlap, or indirectly by elevating the background level of radiation under the peak of interest via Compton events and/or bremsstrahlung [22]. Radiochemical separation can solve also another problem caused by possible spectral interference from radionuclide ^147^Nd with gamma line 319, 4 keV (^51^Cr gamma line 320 keV).

An inorganic ion exchanger MnO_2_ Resin (manganese(IV) dioxide) was chosen for the selective separation of chromium from the biological samples. The sorbent was devised for the separation of radium from barium in natural water samples over a wide range of pH. It was also used to isolate radium from the liquid wastes from the uranium industry and sorption of radionuclides released into sea water from a nuclear reactor [23]. Recently, the resin was applied to the separation of chromium from human blood [24]. As an inorganic resin, it is resistant to high temperature and ionizing radiation, and shows high selectivity to polyvalent metals [25, 26].

In order to find the best condition for quantitative and selective separation of chromium from other elements, mass distribution coefficients of Cr, Zn, Co, Sc, Cs have been determined in the system: MnO_2_ Resin—HCl, HNO_3_ and H_2_SO_4_ in the concentration range from 0.01 to 8 M. The mass distribution coefficients (K_d_) were determined by batch equilibration at room temperature using radioactive tracers of elements in question. The distribution coefficients were calculated from the equation:

1 $${\text{K}}_{\text{d}} = \frac{{{\text{A}}_{0} - {\text{A}}_{\text{S}} }}{{{\text{A}}_{\text{S}} }} \times \frac{\text{V}}{{{\text{m}}_{\text{j}} }}$$

where A_0_ is the concentration of individual tracer in the standard solution; A_S_ is the concentration of individual tracer in an aliquot of the solution, after equilibration with the resin, loaded with a given extractant; V is the volume of the solution (mL); and m_j_ is the mass of dry resin (g). The obtained results are shown in Table 1.

**Table 1:** Distribution coefficients K_d_ of chromium(VI), Cs, Sc, Zn and Co in the system: MnO_2_ Resin—HCl, HNO_3_, H_2_SO_4_

Acid	C (mol L^−1^)	^51^Cr	^134^Cs	^46^Sc	^65^Zn	^60^Co
HCl	0.01	43,147	252	329	13	2246
HNO_3_		7003	175	172	5	965
H_2_SO_4_		79,299	293	93	5.64	1292
HCl	0.1	3668	147	25	12	949
HNO_3_		2241	67	16	5	407
H_2_SO_4_		259	190	0.92	4.89	240
HCl	0.5	1252	76	3	29	181
HNO_3_		436	101	4	12	46
H_2_SO_4_		52	136	0.68	3.65	24
HCl	1	1803	90	13	84	103
HNO_3_		197	72	4	12	17
H_2_SO_4_		35	108	0.52	6.92	14
HCl	2	1043	58	9	313	55
HNO_3_		50	50	4	10	10
H_2_SO_4_		22	63	0.23	4.97	6
HCl	8	6.9	3	4	67	38
HNO_3_		6.2	5	3	11	4
H_2_SO_4_		0.31	10	0.20	2.73	1.64

**Fig. 1:**
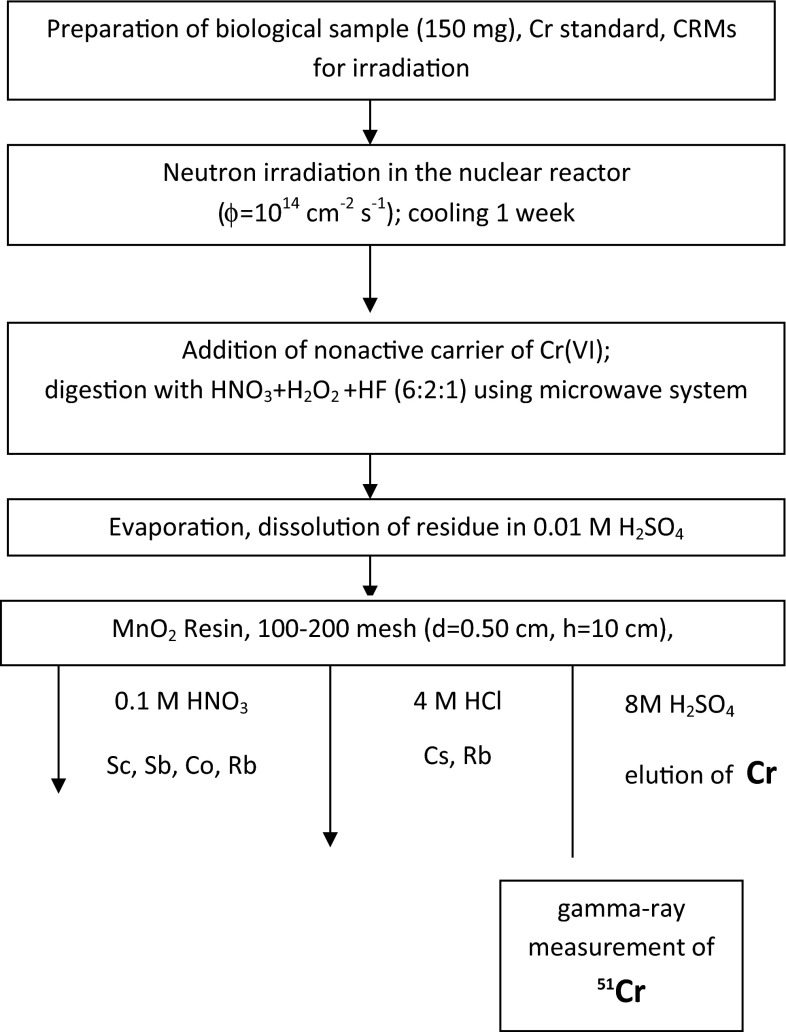
Scheme of the RNAA procedure for determination of Cr in biological materials

**Fig. 2:**
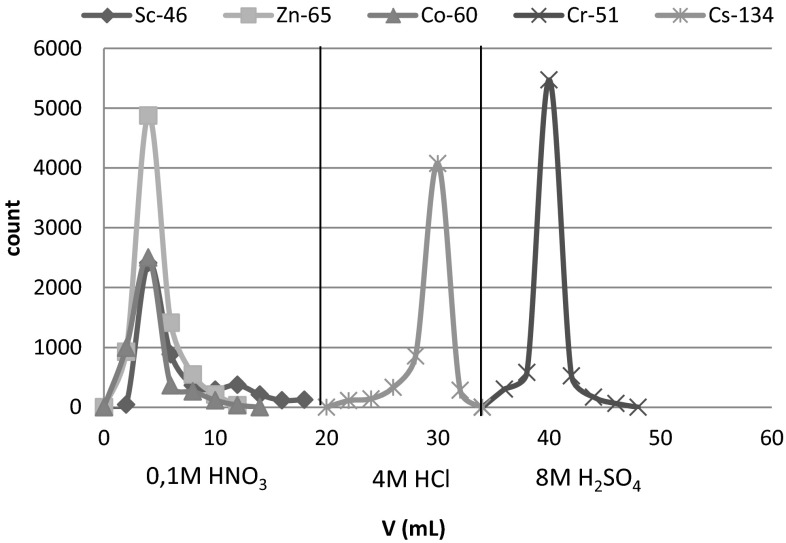
Separation of chromium from selected elements

**Fig. 3:**
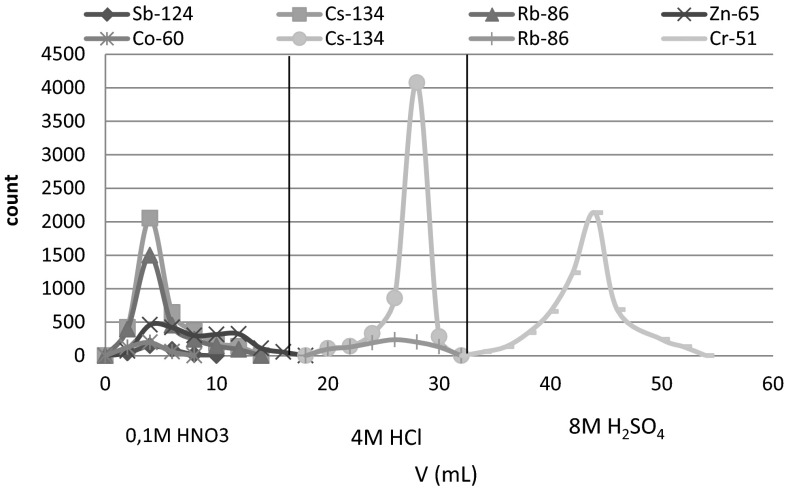
Separation of chromium from other elements—Tea Leaves INCT-TL-1

**Fig. 4:**
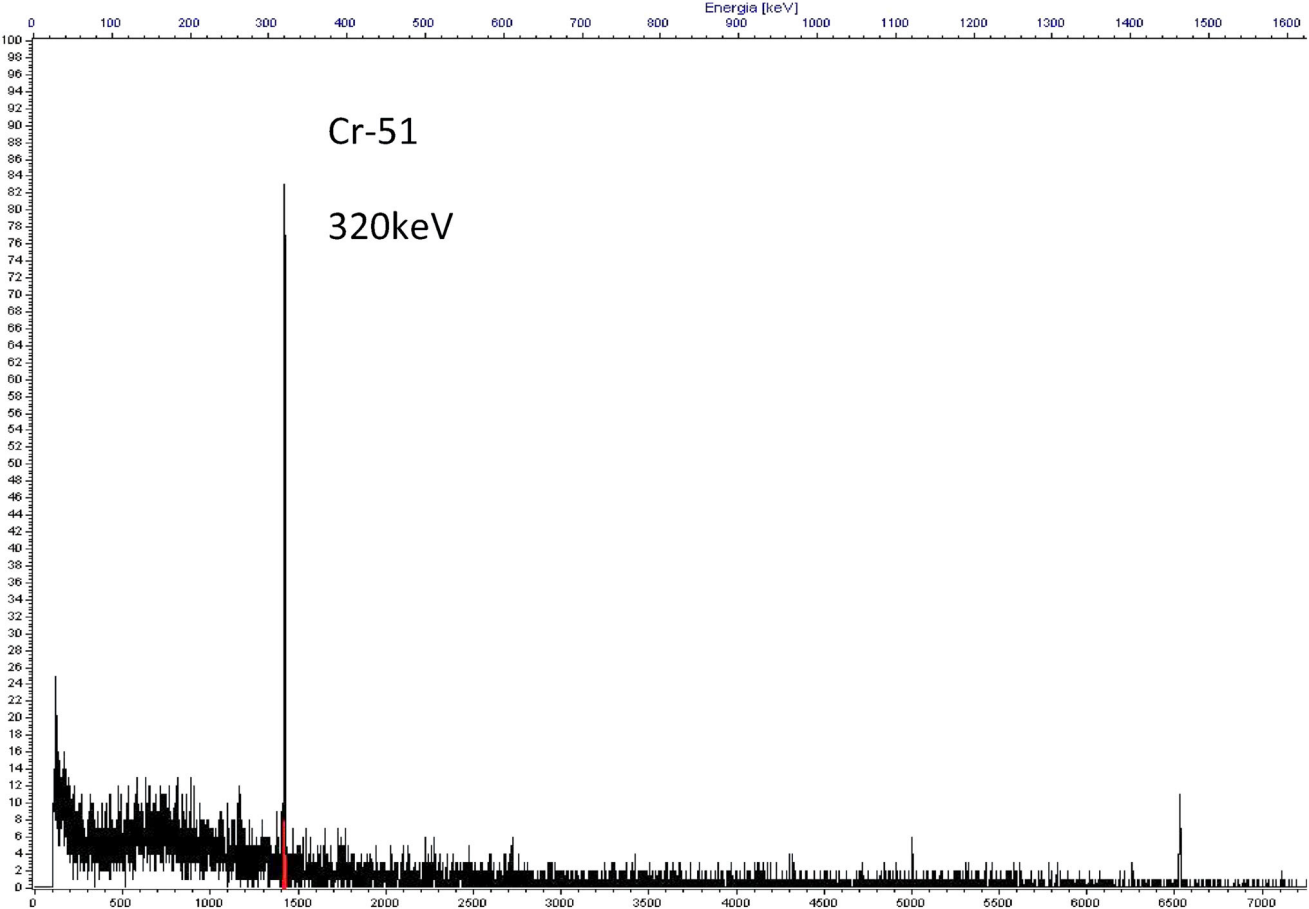
Gamma-ray spectrum of chromium fraction isolated from Tea Leaves INCT-TL-1

As can be seen from Table 1, the absorption of Cr(VI) takes place in the dilute solutions of hydrochloric, nitric and sulfuric acid, and decreases with increasing concentration of the acids. The distribution coefficient values of chromium in 0.01 M acids are sufficiently high to ensure its quantitative retention on the sorbent. Taking into account the distribution coefficients of the other elements in question, the optimal conditions of separation of Cr from other elements have been chosen (Fig. 1) and verified by the series of column experiments (Fig. 2). The separation of chromium on MnO_2_ Resin from Tea Leaves INCT-TL-1 is shown in Fig. 3, and the obtained gamma-ray spectrum of chromium fraction is shown in Fig. 4.

The devised procedure was validated by the analysis of several CRMs: Tea Leaves INCT-TL-1, Scallop Tissue IAEA-452, Spinach Leaves NBS 1570, Oyster Tissue NIST 1566a and Orchard Leaves SRM 1571.

A comparison of the results of chromium determination obtained by the developed procedure with the certified values is shown in Table 2. All analysis were carried out repeatedly (at least twice).

**Table 2:** Results of the determination of chromium in the certified references materials

Certified reference materials (CRM)	Certified value ± uncertainty (mg kg^−1^)	Value obtained by the developed method X ± U (k = 2) (mg kg^−1^)
INCT-TL-1 Tea Leaves	1.91 ± 0.22	2.04 ± 0.09
IAEA-452 Scallop Tissue	5.2 ± 0.70	5.45 ± 0.13
NBS SRM 1570 Spinach Leaves	4.6 ± 0.30	4.37 ± 0.21
NIST SRM 1566a Oyster Tissue	1.43 ± 0.46	1.39 ± 0.05
NBS SRM 1571 Orchard Leaves	2.60 ± 0.30	2.40 ± 0.04

As can be seen from Table 2, the results of Cr determination in biological materials, obtained by the elaborated RNAA procedure agree very well with the certified values. The obtained results confirmed also absence of spectral interferences from ^147^Nd, especially in the case of Tea Leaves INCT-TL-1, which contains relatively high amount of Nd (informative value 0.8 mg kg^−1^). This was to be excepted because K_d_ of scandium was very low in all used acids (Table 1) what indicated that REE should be not retained on MnO_2_ Resin.

The detection limit (LOD) of the procedure was calculated from Currie criterion [27] as 4.88 ng g^−1^ (20,000 s counting time). The combined standard uncertainty of the measurement results was evaluated according to the uncertainty propagation law, taking into account all individual uncertainty sources starting from the weighing of samples and standards, through chemical separation yield and finishing with the gamma-spectrometric measurements [14, 28]. The details of the evaluation of the standard uncertainties associated with individual sources of uncertainty have been described earlier [14–21].

As can be seen from Table 2, the values of the expanded combined uncertainty vary from 1.67 to 4.81 %.

## Conclusions

The elaborated method based on RNAA can be used to the determination of total chromium content in biological materials. Chromium is selectively and quantitatively separated from other elements utilizing the inorganic sorbent MnO_2_ Resin. The described method gives accurate results with low levels of uncertainty. The method can be applied to verify the accuracy of the results of other methods for the determination of chromium in biological materials.
